# Statement of Retraction: Protein tyrosine phosphatase receptor type Z1 inhibits the cisplatin resistance of ovarian cancer by regulating PI3K/AKT/mTOR signal pathway

**DOI:** 10.1080/21655979.2024.2299585

**Published:** 2024-02-20

We, the Editors and Publisher of the journal *Bioengineered*, have retracted the following article:

Peng Wang, Yuanjing Hu, Pengpeng Qu, Ying Zhao, Jing Liu, Jianguo Zhao & Beihua Kong (2022) Protein tyrosine phosphatase receptor type Z1 inhibits the cisplatin resistance of ovarian cancer by regulating PI3K/AKT/mTOR signal pathway, Bioengineered, 13:1, 1931-1941, DOI: 10.1080/21655979.2021.2022268

Since publication, concerns have been raised about the integrity of the data in the article. When approached for an explanation, the authors have been unable to fully address the concerns raised and have not been able to provide their original data in the appropriate format. As verifying the validity of published work is core to the integrity of the scholarly record, we are therefore retracting the article. All authors listed in this publication have been informed.

We have been informed in our decision-making by our editorial policies and the COPE guidelines on retractions.

The retracted article will remain online to maintain the scholarly record, but it will be digitally watermarked on each page as ‘Retracted’.

